# Autophagic effects of *Chaihu* (dried roots of *Bupleurum Chinense DC* or *Bupleurum scorzoneraefolium WILD*)

**DOI:** 10.1186/1749-8546-9-21

**Published:** 2014-09-11

**Authors:** Betty Yuen-Kwan Law, Jing-Fang Mo, Vincent Kam-Wai Wong

**Affiliations:** 1 State Key Laboratory of Quality Research in Chinese Medicine, Macau University of Science and Technology, Macau, China; 2 School of Chinese Medicine, Hong Kong Baptist University, Hong Kong, China

**Keywords:** Autophagy, *Chaihu*, saikosaponin, Chinese Medicine, *qi*

## Abstract

*Chaihu*, prepared from the dried roots of *Bupleurum Chinense DC* (also known as *bei Chaihu* in Chinese) or *Bupleurum scorzoneraefolium WILD* (also known as *nan Chaihu* in Chinese), is a herbal medicine for harmonizing and soothing *gan* (*liver*) *qi* stagnation. Substantial pharmacological studies have been conducted on *Chaihu* and its active components (saikosaponins). One of the active components of *Chaihu*, saikosaponin-d, exhibited anticancer effects *via* autophagy induction. This article reviews the pharmacological findings for the roles of autophagy in the pharmacological actions of *Chaihu* and saikosaponins.

## Introduction

**Figure 1:**
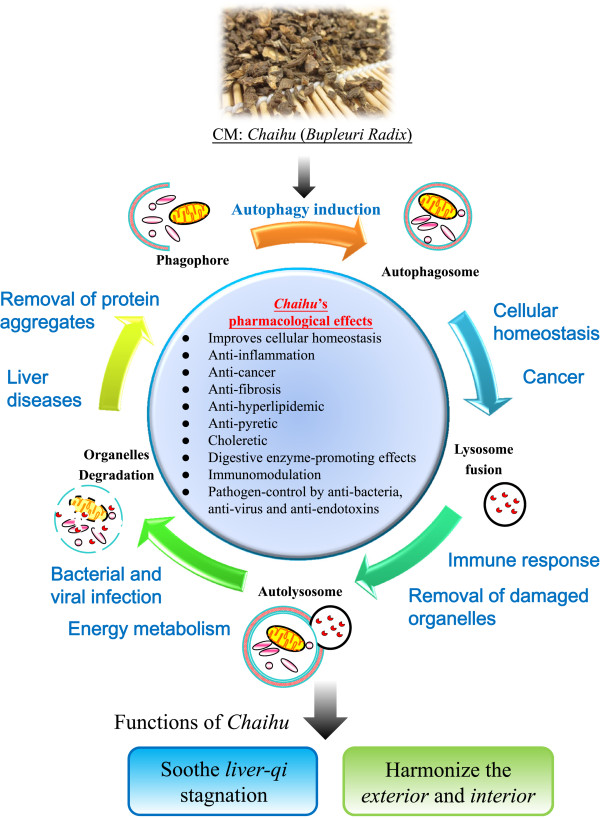
**A schematic diagram illustrating the pharmacological effects of *Chaihu *through autophagy induction.** With its major clinical indications in anti-inflammatory, anticancer, antifibrotic, antihyperlipidemic, and antipyretic functions, *Chaihu* exhibits its pharmacological effects by regulating balanced cellular homeostasis *via* autophagy induction, leading to harmonization and modulation of *qi* in the human body.

*Chaihu*, prepared from the dried roots of *Bupleurum Chinense DC* (also known as *bei Chaihu* in Chinese) or *Bupleurum scorzoneraefolium WILD* (also known as *nan Chaihu* in Chinese), is often prescribed as decoctions such as “*xiao yao* powder”, “*da Chaihu* decoction”, or “*xiao Chaihu* decoction” for treating chills and fevers [1-3]. *Chaihu* facilitates *sheng* (*ascending*) and *jiang* (*dispersing*) *qi* to alleviate stagnation of *gan* (*liver*) *qi*[4]. The contemporary clinical indications for *Chaihu* include common cold, malaria, cholecystitis, globus pharyngitis, gynecological diseases, depression, hepatitis, liver cirrhosis, pancreatitis, and hyperlipidemia [5,6]. Recent research has revealed the pharmacological actions of *Chaihu*. Specifically, *Chaihu* and its active components (saikosaponins) exhibited immunomodulatory [7,8], antiviral [9], antipyretic [10,11], hepatoprotective [12,13], anticancer [14], sedative, and analgesic [15] effects. Our recent study further revealed that saikosaponin-a (Ssa) and saikosaponin-d (Ssd), which are related to *gan qi* regulation [4,13] can induce autophagy [16]. This article reviews the recent findings for the roles of autophagy in the pharmacological actions of *Chaihu* and saikosaponins (Figure 1).

### *Chaihu* regulates *qi* stagnation in Chinese Medicine (CM) theory

The CM approach to relieving symptoms (*e.g.*, physical discomfort and emotional instability) is to soothe stagnation of *gan qi*[17]. *Gan qi* stagnation can lead to (1) distention and pain in the chest and flank, and menstrual dysregulation, (2) impaired digestive functions such as loss of appetite, dyspepsia, flatulence, and regurgitation, and (3) emotional instabilities such as depression, anxiety, and insomnia [18]. *Chaihu* is often prescribed to relieve the symptoms of *qi* stagnation in CM [5].

### Modern pharmacological studies on *Chaihu* and its active components

*Chaihu* alleviates a wide spectrum of disorders in a multi-target manner through its immunomodulatory [7], antipyretic [10], hepatoprotective [13], choleretic [15], autophagy-inducing [16], sedative and analgesic [15], antihyperlipidemic [15], antiviral [9], and anticancer [14] effects.

The pharmacological effects of *Chaihu* are attributed to its active components, Ssa, saikosaponin-c (Ssc), and Ssd [19,20]. Ssa exhibits antiproliferative, anti-inflammatory, anticancer, antioxidative, and hepatoprotective effects [21-26]. Ssc induces umbilical vein endothelial cell proliferation, migration, and capillary vascularization [27], and possesses anti-hepatitis effects [28]. Ssd also exhibits immunomodulatory, antiproliferative, and anticancer effects [29-32]. In particular, Ssd induces autophagy and autophagic cell death in apoptosis-defective cells *via* direct inhibition of sarcoplasmic/endoplasmic reticulum Ca^2+^ ATPase pump (SERCA) and mammalian target of rapamycin (mTOR), with disruption of calcium homeostasis and induction of endoplasmic reticulum (ER) stress [16].

### Autophagy in health and diseases

Autophagy has been highlighted for its protective roles in various physiological and pathological conditions including (1) cellular homeostasis and genome stability maintenance, (2) immunomodulation, (3) hepatoprotection and aggregate removal, (4) cancers, and (5) emotional instability conditions [33-35]. Autophagic regulation is mainly responsible for maintenance of normal cellular and hormonal homeostasis, defense against pathogen invasion, and protection against toxic protein aggregate accumulation, and beneficial improvements in all of these at the cellular level are related to improved *qi* stagnation (Table 1).

**Table 1:** **Comparisons of CM applications, pharmacological actions, and autophagy effects of *Chaihu***

**CM applications**	**Pharmacological effects**	**Autophagic effects**
Improvement of alternating chills and fever	Antipyresis Antibacteria, antivirus, and anti-endotoxin Immunomodulation	Immunomodulation Anti-pathogens Modulation of cytokine secretion Removal of toxic mutant proteins and aggregates
Modulation of inflammatory symptoms and diseases	Immunomodulation Antibacteria and antivirus Modulation of cytokine secretion	Immunomodulation by pathogen and cytokine control Removal of abnormal protein aggregates Detoxification and degradation of toxins and inflammatory proteins
Reduction of distention and pain in the chest and flank Improvement of digestive functions: loss of appetite, dyspepsia, and flatulence	Hepatoprotection Anti-inflammation Anti-fibrosis Promotion of pancreatic digestive enzyme secretion	Cellular catabolism for removal of waste materials Immunomodulation Anti-pathogens Removal of toxic mutant proteins and aggregates Regulation of lipid metabolism
Improvement of circulation or stasis of blood and body fluid, and accumulation of phlegm	Promotion of cancer cell death Reduction in cancer cell proliferation Immunomodulation, apoptosis, and anti-angiogenesis	Maintenance of genomic stability Promotion of autophagic cell death Elimination of damaged proteins and cytotoxic substances
Improvement of emotional instability	Reduction in plasma lipid levels Hormonal regulation Glucose metabolism	Regulation of lipid metabolism Removal of toxic mutant proteins and aggregates

Newborn mice under starvation showed immediate increases of autophagy in various tissues, which returned to the basal levels after nutrient supply restoration [36-38]. Mice deficient in autophagy-related gene (Atg) 5 showed a substantial increase in nutrition deprivation-induced death, suggesting an essential role of autophagy in energy maintenance [39]. Autophagy is a protective mechanism that eliminates abnormal proteins and defective organelles such as mitochondria, peroxisomes, or ER membranes. For example, hepatocytes from Atg7-knockout mice exhibited accumulation of abnormal mitochondria and ER structures [40], and associated cellular degeneration [39]. A recent study further revealed essential roles of autophagy in limiting DNA damage and chromosome instability, and failure of the autophagy process can result in carcinogenesis or cell death [41].

### Chaihu-mediated autophagy induction

Maintenance of normal homeostasis by defense against pathogen infections is critical. Fever is an immune response initiated by inflammatory mediators such as interleukin (IL)-1, IL-6, tumor necrosis factor (TNF)-α, macrophage inflammatory protein 1, and interferon (IFN) for heat production, and depends on antipyretics (IL-10, glucocorticoids, and neuropeptides) for heat dissipation [42,43]. *Chaihu* is prescribed as the major herbal medicine to resolve alternating chills and fever, headache, distention in the chest and flank, or loss of appetite in CM [18,44]. *Chaihu* was reported to exert its antipyretic effect through the thermoregulatory center in the hypothalamus [45]. *Chaihu* inhibited increases in cyclic adenosine monophosphate (c-AMP), an endopyrogen, in the hypothalamus and promoted the release of antipyretic substances [46]. Furthermore, total saikosaponins exerted potent anti-endotoxin effects with a simultaneous reduction in body temperature elevation *in vivo*[47]. All of these beneficial effects can be attributed to the maintenance of cellular homeostasis, a key process regulated by autophagy.

In liver ischemia-reperfusion injury, autophagy induction attenuated the organ damage, and delayed inflammatory or oxidative damage [48]. Furthermore, autophagy suppression was found to be a response to excessive alcohol intake, which might be a reason for the abnormal protein aggregation observed in liver diseases [40]. *In vitro* studies further showed a dysfunction of autophagy in cells with hepatitis C virus infection [48,49]. Autophagy was also found to regulate the immunological responses to invading microorganisms [50]. Another study showed that plasmacytoid dendritic cells recognized viruses *via* Toll-like receptors (TLRs) with a requirement for autophagy [51]. In addition, defective autophagy was involved in inflammatory diseases such as systemic lupus erythematosis and Crohn’s disease [52,53]. Emerging evidence has suggested roles for autophagy in immunological responses including antimicrobial activity, antigen presentation, cytokine production, and regulation of lymphocytes [50,54]. For example, disruption of the virulence factor from the HSV-1 virus, which inhibited the host autophagy proteins, could prevent fatal encephalitis [55]. In addition, autophagy exhibited protective functions in the spleen, bone marrow, or liver through activation of immune responses such as detoxification and degradation of toxins and inflammatory proteins [56-58].

*Chaihu* regulated the immune responses against invading pathogens by stimulating the secretion of glucocorticoids and inhibiting inflammation and anaphylaxis [59,60], and was involved in inflammatory processes such as infiltration, capillary permeability, and release of cytokines [46]. *Chaihu* or its component saikosaponins eliminated exogenous pyrogens through their antibacterial properties [61], and possessed antiviral activities toward hepatitis B [62], human coronavirus 229E [9], influenza virus [11], and respiratory syncytial virus [63]. Ssd reduced the levels of cyclooxygenase and lipoxygenase *in vitro*, promoted IL-2 and IL-4 production, and inhibited IL-6, TNF-α, and IFN-γ expression in mouse T lymphocytes [64,65]. The prominent anti-inflammatory effects of *Chaihu* could be mediated through autophagy induction, a key process for pathogen elimination and immunity regulation. Our group was the first to report the autophagic activities of *Chaihu* and Ssd [16]. We hypothesized that *Chaihu* harmonizes the exterior and interior of the human body and soothes *gan qi* stagnation through autophagy induction.

### Chaihu-induced autophagy alleviates gan qi stagnation

In CM theory, *Chaihu* soothes stagnation of *gan qi* and promotes circulation of *qi*, and thus alleviates distention and pain in the chest and flank, menstrual dysregulation, impaired digestive functions such as loss of appetite, dyspepsia, flatulence, and regurgitation, and emotional instabilities such as depression, anxiety, and insomnia [18]. *Chaihu* is used to treat diseases related to the digestive system, *e.g.*, hepatitis, liver cirrhosis, cholecystitis, pancreatitis, gynecological diseases, and hyperlipidemia [5].

Saikosaponins alleviated hepatocytes from oxidative and inflammatory stresses, and inhibited liver fibrosis [66]. Further studies demonstrated the protective effects of saikosaponins in reducing lipid peroxidation in hepatocytes [67], regulating intracellular calcium levels to prevent hepatocyte injury [68], suppressing activation of hepatic stellate cells as the major matrix-producing cells in liver fibrosis [69,70], and reducing collagen I deposition in the rat liver [71]. Saikosaponins exhibited regulatory effects on cytokines such as ILs, TNF, and IFN [64,65], inhibitory effects on infiltration of macrophages and T lymphocytes [72], and bidirectional modulation of splenic T lymphocyte proliferation [64]. These findings suggest that the hepatoprotective effects of *Chaihu* and saikosaponins are related to improvement of *gan qi* stagnation. In addition to liver diseases, *Chaihu* is commonly used for chronic pancreatitis [73]. Saikosaponins exhibited potent stimulatory effects on pancreatic enzyme secretion in rats [74]. *Chai-hu-shu-gan* powder inhibited the expression of nuclear factor-κB (NF-κB) and TNF-α mRNA in the pancreas to achieve anti-inflammatory and antifibrotic effects [75]. Moreover, the same prescription reduced the abnormally high plasma level of cholecystokinin in chronic pancreatitis, improved the gastric movement, and avoided nausea and flatulence [76,77].

In liver ischemia-reperfusion injury, autophagy induction attenuated the ischemic and reperfusion damage to the organ, probably because a decrease in autophagy would lead to accumulation of dysfunctional mitochondria, resulting in cellular damage and failure in energy production, and eventually cell death [48]. In liver disease, suppression of autophagy caused abnormal protein aggregation [40]. In liver fibrosis, autophagy activation might be beneficial to the recovery of the liver function [78]. All of these findings indicate that *Chaihu*-induced autophagy might relieve liver disease-related symptoms through anti-inflammatory, organ-protective, and aggregate removal functions, which are related to alleviation of *gan qi* stagnation.

### Chaihu-mediated autophagy intervenes in carcinogenesis

In CM theory, tumor formation is the result of stasis of *xue* (*blood*), retention of *jin ye* (*fluid*), and accumulation of *tan* (*phlegm*) [79]. A recent study demonstrated the anticancer effects of Ssa and Ssd *via* autophagy induction and autophagic cell death [16]. In addition, *Chaihu* is a commonly prescribed herb in contemporary formulations (Table 2) with preventive or therapeutic effects on cancer [80]. Patients treated with “*xiao Chaihu*” decoction exhibited a significantly lower incidence of hepatocellular carcinoma [81], reductions in cancer pain and tumor size [82,83], and prevention of liver cancer relapses [84]. The decoction had multiple functions in immunomodulation, apoptosis, and anti-angiogenesis [85-87].

**Table 2:** ***Chaihu*-containing formulated decoctions prescribed for modulation of cancers in CM**[80]

**Cancer**	***Chaihu*-containing prescriptions**
Hepatocellular cancer	*Xiao Chaihu* Decoction
	Supplemented *Da Chaihu* Decoction
	*Si ni* Powder combined with *Liu jun zi* Decoction
	Supplemented *Xiao yao* Powder
	No. 1 anticancer formula
	*Chaihu zhe chong* Decoction
	Experienced prescription
Pancreatic cancer	*Xiao Chaihu* Decoction
	Experienced prescription
Gall bladder cancer	*Shu gan li dan* Decoction
Breast cancer	*Yi qi shu gan* Decoction
	*Xiao ru* Decoction
	Supplemented *Xiao yao* powder combined with *Si jun zi* Decoction
	Experienced prescription
Cervical cancer	*Jia wei xiao yao* Powder
	*Chaihu gui zhi* Decoction
Thyroid carcinoma	*Jia xian ping* Decoction
Esophageal carcinoma	*Jin fo yin*
	*Er chen xuan fu* Decoction
Gastric cancer	*Chaihu shu gan* Decoction combined with *Xi shu jian*

The signaling pathway of autophagy is associated with the key regulatory proteins of carcinogenesis, such as tumor suppressor gene p53, phosphatase and tensin homolog (PTEN), death-associated protein kinase, and proto-oncoprotein B-cell CLL/lymphoma 2 (Bcl-2) [39,88]. Autophagy was responsible for massive cancer cell death *in vitro* and *in vivo*[89-91]. Autophagic inducers also promoted autophagic cell death in tumors or augmented the efficacy of chemotherapeutic agents when used in combination during cancer therapy [92,93]. By eliminating genomic mutations, damaged proteins, and cytotoxic substances, autophagy protected cells against cancers [94]. However, the roles of autophagy in cancers remain controversial, because autophagy might promote tumor growth by providing energy to poorly-vascularized tumor cells [95].

Despite its adaptive and pro-survival roles, autophagy can lead to type II programmed cell death [96]. Autophagy promoted autophagic cell death in cells [97], and killed apoptosis-resistant cancer cells under chemotherapy [98]. Moreover, autophagy was associated with massive cancer cell death in cancerous tissues derived from different organs [99,100]. Ssd was able to induce autophagic cell death in a panel of apoptosis-resistant cells *via* direct inhibition of SERCA [16]. The anticancer effects of *Chaihu* can be attributed to its autophagy-inducing ability.

### Chaihu-mediated autophagy modulates stress hormone-regulated metabolism

*Chaihu* could mediate its protective effects on *gan qi* stagnation-induced emotional instability through lipid metabolism and hormonal regulation [101]. In fact, analyses of plasma metabolites in a rat model of *gan qi* stagnation stimulated by chronic immobilization stress revealed elevated levels of lactic acid, saturated fatty acid, and blood sugar, and reduced levels of unsaturated fatty acid and high density lipoprotein [102]. Another study applied stress to a macaque model with premenstrual syndrome, and demonstrated increased plasma levels of serotonin (5-HT), noradrenalin, and prolactin [103].

As a regulator of lipid and glucose metabolism [104], loss of autophagy caused abnormal accumulation of lipids in mouse hepatocytes and a significant increase in plasma triglycerides, with reductions in fatty acid beta-oxidation [105] and pancreatic β-cell mass [106]. Coincidently, saikosaponins increased hepatic uptake of cholesterol and decreased plasma levels of cholesterol and triglycerides [107]. Furthermore, a study on depressive patients revealed correlations between the plasma levels of cholesterol, triglycerides, and serum neurotransmitters, and depression [108]. As saikosaponins were able to reduce the plasma levels of cholesterol, triglycerides, and phospholipids [107], *Chaihu* might attenuate depressive symptoms by regulating metabolite, hormone, and neurotransmitter levels *via* autophagy-mediated lipid metabolism in the human body.

## Conclusions

The function of *Chaihu* in harmonizing the exterior and interior of the body is related to its pathogen control and immunomodulation properties. Furthermore, *Chaihu*’s function in resolving *gan qi* stagnation might arise through its supportive roles in protecting organs, preventing damage to cells and organs, and restoring visceral and cellular metabolic conditions. All of these protective pharmacological effects of *Chaihu* might be attributed to its autophagy induction.

## Abbreviations

Ssa: Saikosaponin-a; Ssc: Saikosaponin-c; Ssd: Saikosaponin-d; ER: Endoplasmic reticulum; PTEN: Phosphatase and tensin homolog; TLRs: Toll-like receptors; Bcl-2: B-cell CLL/lymphoma 2; IL: Interleukin; TNF: Tumor necrosis factor; IFN: Interferon; c-AMP: Cyclic adenosine monophosphate; SERCA: Sarcoplasmic/endoplasmic reticulum calcium ATPase pump; NF-κB: Nuclear factor-κB; CM: Chinese medicine; Atg: Autophagy-related gene; mTOR: Mammalian target of rapamycin; 5-HT: 5-hydroxytryptamine.

## Competing interests

The authors declare that they have no competing interests.

## Authors’ contributions

VKWW conceived and planned the review. BYKL and JFO carried out the review plan and wrote the manuscript. All authors read and approved the final manuscript.
